# A multiple-response frequency-tagging paradigm measures graded changes in consciousness during perceptual filling-in

**DOI:** 10.1093/nc/niaa002

**Published:** 2020-04-12

**Authors:** Matthew J Davidson, Irene L Graafsma, Naotsugu Tsuchiya, Jeroen van Boxtel

**Affiliations:** n1 School of Psychological Sciences, Faculty of Medicine, Nursing and Health Sciences, Monash University, Melbourne, Australia; n2 Department of Psychology, University of Amsterdam, Amsterdam 1001 NK, the Netherlands; n3 Department of Cognitive Science, Macquarie University, Sydney, Australia; n4 Center for Language and Cognition Groningen (CLCG), University of Groningen, the Netherlands; n5 Turner Institute for Brain and Mental Health, Faculty of Medicine, Nursing and Health Sciences, Monash University, Melbourne, Australia; n6 Center for Information and Neural Networks (CiNet), National Institute of Information and Communications Technology (NICT), Suita, Osaka 565-0871, Japan; n7 Advanced Telecommunications Research Computational Neuroscience Laboratories, 2-2-2 Hikaridai, Seika-cho, Soraku-gun, Kyoto 619-0288, Japan; n8 Department of Psychology, Faculty of Health, University of Canberra, Canberra, Australia

**Keywords:** contents of consciousness, methodology, perception, psychophysics

## Abstract

Perceptual filling-in (PFI) occurs when a physically present visual target disappears from conscious perception, with its location filled-in by the surrounding visual background. These perceptual changes are complete, near instantaneous, and can occur for multiple separate locations simultaneously. Here, we show that contrasting neural activity during the presence or absence of multi-target PFI can complement other findings from multistable phenomena to reveal the neural correlates of consciousness (NCC). We presented four peripheral targets over a background dynamically updating at 20 Hz. While participants reported on target disappearances/reappearances via button press/release, we tracked neural activity entrained by the background during PFI using steady-state visually evoked potentials (SSVEPs) recorded in the electroencephalogram. We found background SSVEPs closely correlated with subjective report, and increased with an increasing amount of PFI. Unexpectedly, we found that as the number of filled-in targets increased, the duration of target disappearances also increased, suggesting that facilitatory interactions exist between targets in separate visual quadrants. We also found distinct spatiotemporal correlates for the background SSVEP harmonics. Prior to genuine PFI, the response at the second harmonic (40 Hz) increased before the first (20 Hz), which we tentatively link to an attentional effect, while no such difference between harmonics was observed for physically removed stimuli. These results demonstrate that PFI can be used to study multi-object perceptual suppression when frequency-tagging the background of a visual display, and because there are distinct neural correlates for endogenously and exogenously induced changes in consciousness, that it is ideally suited to study the NCC.

## Introduction

In perceptual filling-in (PFI) phenomena, areas of the visual environment that are physically distinct become interpolated by the visual features of the surrounding texture or background (Ramachandran and Gregory 1991; Pessoa *et al.* 1998; Meng *et al.* 2005; Komatsu 2006; Weil and Rees 2011). Although PFI neatly displays how our awareness of a visual scene is shaped by (unconscious) inferential processes (Komatsu 2006), it has traditionally been investigated to understand how our visual system compensates for retinal-blind spots (Ramachandran and Gregory 1991; Durgin *et al.* 1995; Komatsu *et al.* 2000; Spillmann *et al.* 2006) and visual field defects (Gassel and Williams 1963; Gerrits and Timmerman 1969; Safran and Landis 1996). Accordingly, a range of low-level visual attributes such as target contrast (Stürzel and Spillmann 2001), target eccentricity (De Weerd *et al.* 1998), and microsaccades (Martinez-Conde *et al.* 2006) have been investigated as potential causes that affect the phenomenal properties and dynamics of PFI. In this context, the neural interpolation of information in lower visual areas has been implicated as one active mechanism behind PFI (De Weerd *et al.* 1995; Pessoa *et al.* 1998; Meng *et al.* 2005; Komatsu 2006). Here, we were motivated to explore the usefulness of PFI as a method to capture changes in conscious awareness in a graded manner, and its potential to contribute to ongoing investigations of the neural correlates of consciousness (NCC). 

We believe PFI can contribute to NCC research for two main reasons. Firstly, PFI spontaneously occurs with prolonged fixation, similar to other multistable phenomena, and can occur over multiple regions embedded in the same visual background (Lou 1999; De Weerd 2006; De Weerd *et al.* 2006). It may therefore be possible for neural measures to capture graded changes in awareness when more than one stimulus simultaneously disappears. This feature may enable future studies to test theoretical accounts of whether neural measures of perception correlate with a gradual or all-or-none phenomenon (Sergent and Dehaene 2004; Tagliabue *et al.* 2016; Haque *et al.* 2019), and to reduce the requirements for tracking changes in awareness via motor-report (Tsuchiya *et al.* 2015). Secondly, although not tested in the present manuscript, selectively attending to the location of a target or the shared features among peripheral targets (Lou 1999; De Weerd 2006; De Weerd *et al.* 2006) also increases the likelihood of PFI. Thus, PFI could provide us a rare opportunity, where top-down attention and conscious perception appear to result in opposing effects, and therefore provide key answers to one hotly debated issue in consciousness research: the nature of the relationship between attention and consciousness (Iwasaki 1993; Posner 1994; Velmans 1996; Hardcastle 1997; Lamme 2003; Dehaene *et al.* 2006; Koch and Tsuchiya 2007; van Boxtel *et al.* 2010); and for recent reviews see Special Issues in *Frontiers in Psychology*—(Tsuchiya and Van Boxtel 2013) and in *Philosophical Transactions*—(Fazekas and Overgaard 2018).

To demonstrate the potential of PFI in this line of research, we first sought to capture neural markers of changes in awareness with a multi-target and multi-response paradigm. To capture the neural correlates of these changes, we combined multi-target PFI with frequency-tagging in the electroencephalogram (EEG) (Vialatte *et al.* 2010; Norcia *et al.* 2015). Only one previous study has investigated PFI using frequency-tagged responses, and flickered only a single target in their display (Weil *et al.* 2007). Here we frequency-tagged the background of our visual display, and analysed response-locked changes to *background* activity. We hypothesized that as the phenomenological interpolation of target regions increased, an increased neural response to the surrounding visual background would be recorded. To foreshadow our results, frequency-tagging the background showed graded responses to the amount of change in conscious perception. With this paradigm, we were also able to distinguish the neural correlates of PFI from phenomenally matched disappearances (PMD), and reveal novel spatiotemporal profiles of frequency-tagged responses which prompt further research using our novel paradigm. Therefore, we suggest that the combination of PFI and frequency-tagging can be a valuable addition in search of the NCCs.

## Materials and Methods 

### Participants

Twenty-nine healthy volunteers were recruited for this experiment. As the effect size of frequency-tagging the background stimuli is unknown, we chose to test 50% more participants than previous studies that used frequency-tagging to study target responses during PFI (Weil *et al.* 2007), or the neural correlates of perception during binocular rivalry (e.g. Lawwill and Biersdorf 1968; Brown and Norcia 1997; Tononi *et al.* 1998; Srinivasan *et al.* 1999; Cosmelli *et al.* 2004; Srinivasan 2004; Srinivasan and Petrovic 2006; Kamphuisen *et al.* 2008; Sutoyo and Srinivasan 2009; Zhang *et al.* 2011; Katyal *et al.* 2016). Participants had normal or corrected-to-normal vision. All participants were recruited via convenience sampling, provided written informed consent prior to participation and received a monetary compensation (30 Australian Dollars) for their time. The study was approved by the Monash University Human Research and Ethics Committee (MUHREC #CLF016). We excluded seven participants due to low quality behavioural data, or due to not reporting any PFI during testing, bringing our final sample to *N *=* *22 (13 female, 18–39 years of age, *M *=* *24.6, SD =5.4 years).

### Apparatus and stimuli

Participants were seated in a dark room approximately 50 cm distance from a computer screen (size 29 × 51 cm, resolution 1080 × 1920 pixels, subtending 32 × 54° visual angle, refresh rate 60 Hz). We did not stabilize head position via chin-rest, and so minor movements and adjustments to visual angle may have occurred. To frequency-tag the background image, we prepared 100 random patterns prior to the start of each experiment by first downsampling the screen to 540 × 960 pixels. We then assigned a random luminance value (drawn from a uniform distribution from black to white) to each down-sampled pixel. These background images were refreshed at a rate of 20 Hz by randomly selecting from the set of 100 prepared patterns.

**Figure 1.**
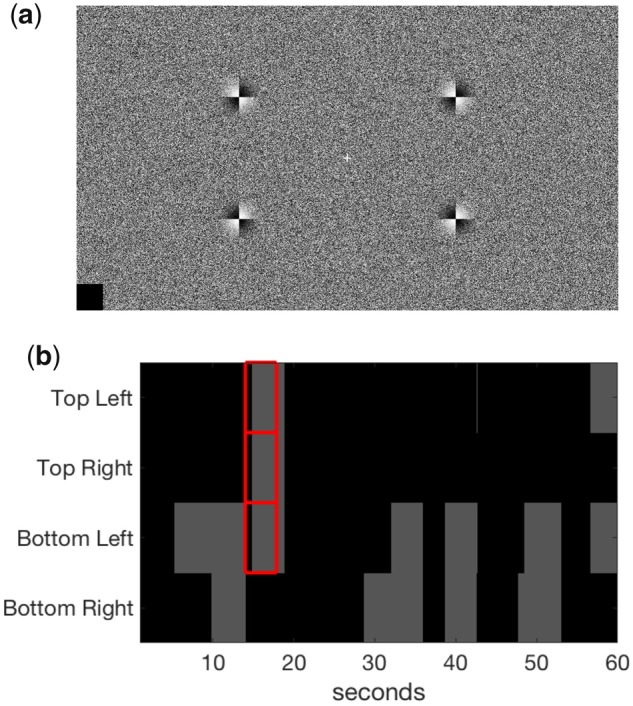
Stimulus display and example response. (**a**) Stimulus display containing a central fixation cross, dynamic background (updated at 20 Hz) and four target checkerboard stimuli. (**b**) Example time course of behavioural responses over a 60-s trial from one participant. Participants were asked to press and hold each button upon perceptual disappearance (PFI events shown in grey) at the corresponding location of the target. Phenomenally matched disappearances (PMD) are shown in red, for which targets were physically replaced by the flickering background texture. An example trial of this display can be viewed at https://osf.io/uwxeh/.

On top of this background image, the display was composed of a central fixation cross (1.03° visual angle in height and width), surrounded by four counter-phase flickering 2 × 2 checkerboard targets (4.56° visual angle in diameter). The checkerboard elements of these targets reversed polarity every half-cycle of an approximately sinusoidal envelope, approximating one of four unique frequencies (8, 13, 15 and 18 Hz). Most of these sinusoidal envelopes were not an exact divisor of our monitor’s refresh rate, but still have the most power at the chosen frequencies. We chose these frequencies, because small, peripherally located targets are more likely to disappear when flickering above 7 Hz (Schieting and Spillmann, 1987; Anstis, 1996). A target was located in each quadrant, centred at 23.3° eccentricity from the centre of the screen. The targets were located closer to the horizontal than vertical: horizontal distance from centre 20.2°, vertical distance from centre 11.5° (Fig. 1). Targets were smoothly alpha-blended into the background texture following a 2D Gaussian profile (SD = 1.06° visual angle in diameter). We chose to use small, peripherally located flickering targets to optimally induce PFI, yet these same parameters are sub-optimal for frequency-tagging stimuli. As a result, our simultaneous background flicker, which is the novel element of our experiment, remains as a relatively pure EEG measure for tracking the disappearance of multiple PFI targets.

### Task procedure

Each experimental session was composed of 25 trials, 60 seconds per trial. Between the trials, participants were able to take short self-timed breaks, resulting in a total time-on-task of approximately 30 minutes. Participants were instructed to fixate on the central cross, and informed they may sometimes experience a visual illusion where any number of peripheral targets disappear from their field of vision. We did not monitor eye position. Participants then completed one practice trial to familiarize themselves with the four button presses required for targets in each visual quadrant. Specifically, they were instructed to press keys ‘A’, ‘Z’, ‘K’ and ‘M’ on a traditional QWERTY keyboard, assigning them to the upper left, bottom left, upper right and bottom right targets, respectively. Participants were instructed to hold each button for the duration of disappearance, and to release it immediately upon the corresponding reappearance of the target. Figure 1 presents the basic configuration of the experimental display used.

### Phenomenally matched disappearances

We introduced phenomenally matched disappearances (PMD) without informing participants, to check if participants were correctly reporting on perceptual disappearances and maintaining attention on task. These physical PMD also served as a control condition for comparison with the neural signals evoked by PFI.

During a PMD, one to four targets were physically removed from the display and replaced with the background by linearly ramping the alpha blending value of the target from 1 to 0 (or vice versa) over 1.5 seconds. Each PMD (without ramps) was 3.5–5 seconds in duration (drawn from a uniform distribution). When two or more targets were removed, their disappearance was synchronous. The location of the removed targets in the case of one, two and three targets were randomized (each occurring on six trials per participant). The order of these PMD events were also randomized for each participant. To mimic the overall dynamics of PFI (Schieting and Spillmann, 1987), we did not include PMD within the first 10 seconds of each trial. We also did not include PMD within the last 10 seconds. Four-target PMD occurred only for 7 of our final 22 participants due to a coding error, resulting in PMD being presented on 83% of trials overall.

### Participant and trial exclusion based on PMD

**Figure 2.**
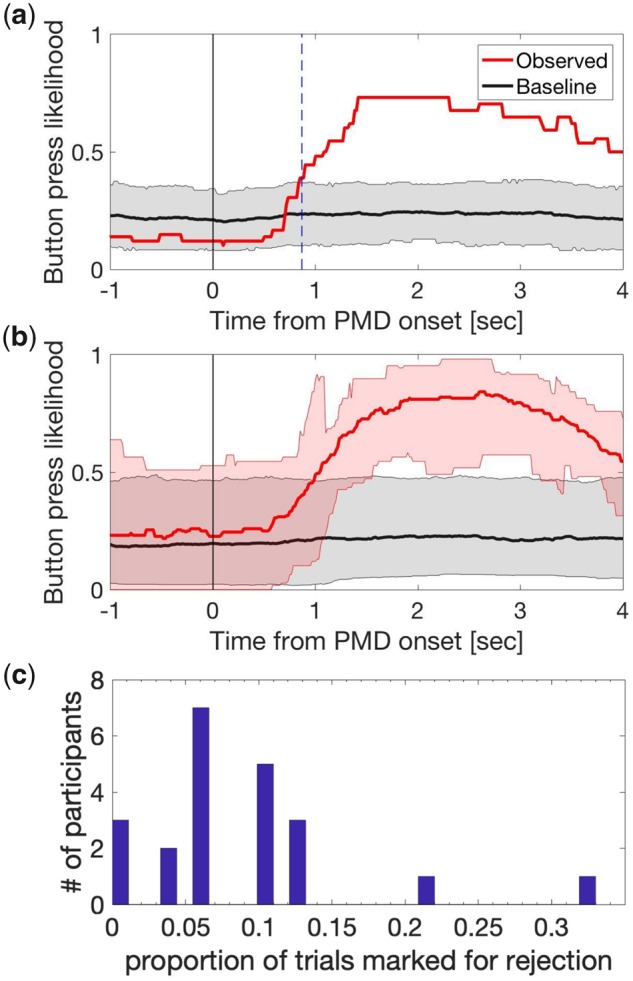
PMD analysis and trial rejection following the physical removal of flickering targets at PMD onset. (**a, b**) Display the likelihood of button-press time-courses for observed (red) and bootstrapped data (grey). (a) Example PMD response for a single participant. The first time point that the observed likelihood of button press (red) exceeded the bootstrapped CI (grey) corresponds to the PMD reaction time (0.87 seconds for this participant, marked with a vertical dashed blue line). (b) The mean time course for the likelihood of button press and its bootstrapped sets across participants, shown in red and grey, respectively. Shading represents the CI (computed with logit transform and presented after reverse transform) across participants. (**c**) Participant-level histogram of the proportion of trials rejected, based on period-by-period PMD analysis.

Initial screening analyses sought to confirm whether participants were able to simultaneously monitor the visibility of multiple peripheral targets using four unique buttons and perform this task accurately in compliance with instructions. Due to a keyboard malfunction, button-press responses to three and four disappearing targets became indistinguishable in our *post hoc* analysis. Thus, we analysed the state of three button press as ‘3 and 4 buttons pressed’. We analysed the likelihood of button-press responses during PMD to estimate participant’s attention on task. As PMD were embedded within a trial, some PMD occurred when participants had already pressed buttons. Such events are more frequent for those who report more frequent PFI, and accounting for this baseline likelihood of button-press is necessary to accurately estimate PMD responses. To estimate this baseline button-press rate per individual participant, we performed a bootstrapping analysis with replacement. For a given PMD onset in trial T at time S (seconds), we randomly selected a trial *T*′ (*T* = *T*′ was allowed) and epoched the button-press time course over the period of [*S* − 2, *S* + 4] at corresponding PMD target locations in trial *T*′. We repeated this for all trials (*T* = 1…24, except for the 4-PMD error mentioned above) to obtain a single bootstrapped set of trials per participant. We then obtained the mean button-press time course across button-locations from this bootstrapped set, and repeated the procedure 200 times to obtain a null distribution, representing the likelihood of baseline button-press around PMD onset per participant (Fig. 2a, grey lines and shading). We also obtained the mean button-press time course for observed data across button-locations.

As the distribution at each time point for both observed and baseline data was not normally distributed, we first converted the data into *z*-scores using the logit transformation before calculating the confidence intervals (CI). Then, we used mean *z*-scores (±1.96 SD of *z*-scores) as the CI for the null distribution of baseline data within each participant, and observed data across participants.

We excluded three participants whose mean button-press time course around the actual PMD onset failed to exceed the CI of the baseline button-press likelihood within the first 2 seconds (i.e. [0, *S* + 2]). We defined the PMD-onset reaction time as the first time point after which the mean buttonpress data exceeded the top CI, indicating successful button presses for PMD targets. Figure 2a shows the PMD response for an example participant retained for analysis. Four further participants were removed from subsequent analyses for failing to experience PFI during most of the experimental session (i.e. only brief events on one or two trials). For the remaining participants, the mean reaction time to respond to PMD onsets, and thus the disappearance of a peripheral target was 0.92 seconds (SD = 0.046). Figure 2b shows the proportion of button-press responses for all PMD events across participants retained for analysis (*N *=* *22). For the corresponding analysis for reappearances, see Supplementary Materials.

Having identified which participants could successfully indicate target disappearance based on their button-press data, we continued to identify and remove any trials from the subsequent analysis in which a PMD was not correctly detected. We undertook this procedure to assure that in all retained trials participants paid proper attention on task and accurately reported on PFI. We regarded a PMD as being successfully identified if participants pressed the corresponding button for at least 50% of the allowed response time window. For multi-target PMD, we applied the same criteria for each button separately. If any button was not pressed at least 50% of the time, the PMD was considered undetected. For four-target PMD, we analysed it as if it was a three-target PMD. This window was from the onset of the PMD plus 1 second (in consideration of the reaction time delay) to the end of PMD. For example, if the PMD under consideration was 3.5 s in duration, we defined the allowed time window to be [1, 3.5] seconds from the PMD onset. Figure 2c shows a participant-level histogram for the proportion of rejected trials (*M ±* SD*:* 1.75 *±* 1.48 trials or 8.96 *±* 7.89% of all trials).

### Quantifying PFI and PFI location-shuffling analysis

We analysed the number of PFI events, average duration of PFI events and total duration of PFI per 60 second trial. Although these variables may be correlated, they have also been shown to reveal complementary data features in similar multi-target designs (Bonneh *et al.* 2001; Thomas *et al.* 2017; McEwen *et al.* 2020). We note that button-press responses to three and four disappearing targets became indistinguishable in our *post* *hoc* analysis, due to a keyboard malfunction, and have been grouped in our analysis as ‘3 and 4 buttons pressed’. Thus, each of these PFI measures were compared based on the number of PFI (nPFI; 1, 2, 3 and 4), quantified by the number of simultaneous button-press responses at each time point. Because transient button-press periods (< 200 ms) could reflect genuine transient percepts, we opted not to exclude these responses from our reported analyses. Having said that, excluding them did not significantly alter the data.

**Figure 3.**
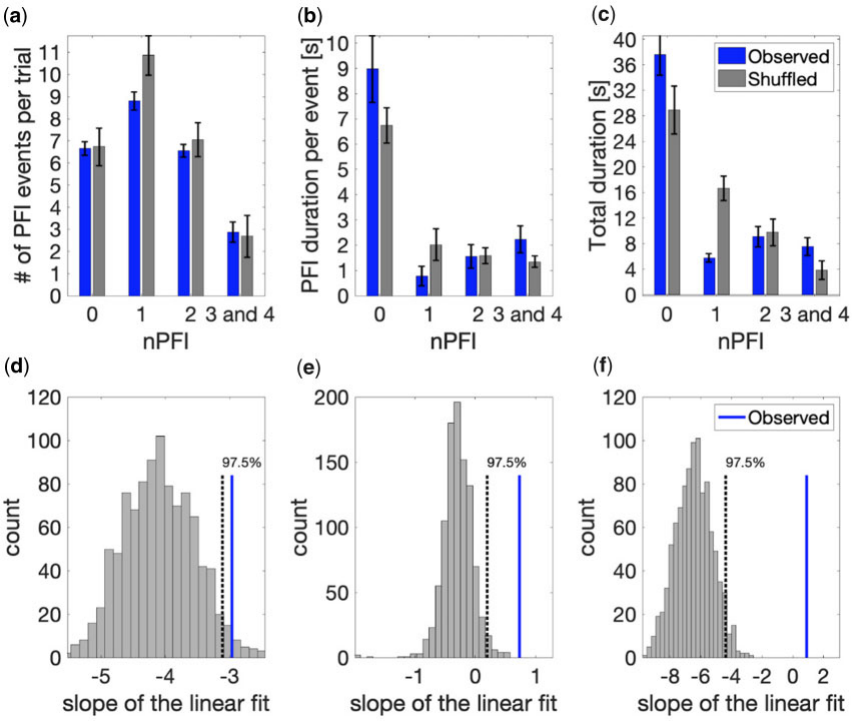
Behavioural data analysis: synergistic disappearance of targets across quadrants. (**a**) The number of PFI events per trial, (**b**) the mean duration per PFI event and (**c**) total duration of PFI per trial, as a function of the number of invisible targets (nPFI). All panels display both observed (blue) and shuffled (grey) data. For the observed data, error bars represent 1 SEM, corrected for within-participant comparisons (Cousineau 2005). For the shuffled data, we first computed the SEM within each shuffled data set across participants. Then, as the error bar, we show the mean of the SEM across 1000 shuffled sets. (**d–f**) Slope of the linear fit for each of the PFI variables in a–c as a function of nPFI for the observed (blue line) vs. the shuffled data (1000 sets, grey histogram).

To investigate whether the simultaneous multi-target PFI observed in participant data (e.g. Fig. 1b) exceeded that expected by chance, we performed a shuffling analysis. We created 1000 shuffled trials for each participant, by randomly selecting the button-press time course for each of the four target locations, independently, from any of the trials throughout their experimental session (this could include multiple locations within the same trial). As the location of each button press in shuffled data could come from any independent trial (e.g. top left = trial 1, top right = trial 23, bottom left = trial 18, bottom right = trial 18), this shuffling procedure conserved the mean number of PFI events overall, while estimating the level of simultaneous invisibility between multiple PFI targets that occurs by chance, when locations are independent. As such these newly created trials provided a null distribution to compare to the observed data, as the presence of a temporal correlation between target locations was removed in shuffled data. The comparison between the observed and the shuffled data is displayed in Fig. 3.

### EEG acquisition and preprocessing

EEG acquisition and preprocessing were performed according to standard procedures (see Supplementary Materials). After examining the topography of log(SNR) responses (see below), we applied rhythmic entrainment source separation (RESS) to optimally extract the time-course of frequency-tagged components of steady-state visually evoked potentials (SSVEPs) without relying upon electrode channel selection (Cohen and Gulbinaite 2016). As RESS produces a single component time-series which is the weighted response from across all channels, we did not use RESS-filtered data for the spatial correlation analysis described below. A more detailed explanation of RESS, and our application, is contained in the Supplementary Materials.

### SSVEP signal-to-noise ratio calculation

To estimate the strength of SSVEPs, we first calculated the natural log of the power spectrum via the fast Fourier transform. In the SSVEP paradigm, we operationally regard power at the tagged frequency as signal and power at non-tagged neighbouring frequencies as noise (Norcia *et al.* 2015) and compute the signal-to-noise ratio (SNR) at each frequency. In logarithmic scale, this corresponds to log of the power at each frequency subtracted by the mean log power across the neighbourhood frequencies. In this article, all SNR results are based on this log-transformed SNR metric because without log-transform, SNR is highly skewed and not appropriate for various statistical tests.

We quantified the topography of SSVEP responses, over the period −3000 to −100 ms before, and 100–3000 ms after button press. We excluded the 200 ms around button-press responses to avoid motor-related activity. Over the 2.9 second time window (half-bandwidth = 0.35 Hz), we computed the SNR at frequency f (Hz) as the mean log power over the neighbourhood frequencies defined as [f − 1.22, f − 0.44] Hz and [f + 0.44, f + 1.22] Hz. In addition, we also computed the time-course of the SNR over a 1 second window (half-bandwidth = 1 Hz) with a step-size of 150 ms, to enable the comparison of fluctuations in SNR over time. For this shorter time window, we used the neighbourhood as [f − 3.92, f − 1.95] Hz and [f + 1.95, f + 3.92] Hz to compute the log(SNR) time course. Our main analyses focus on the time-course of log(SNR) activity during changes in perception. For whole-trial SSVEP power, log(SNR) spectra, and SNR time-course data cleaning see Supplementary Materials.

### Quantifying changes in SSVEP-SNR during changes in the amount of PFI

**Figure 4.**
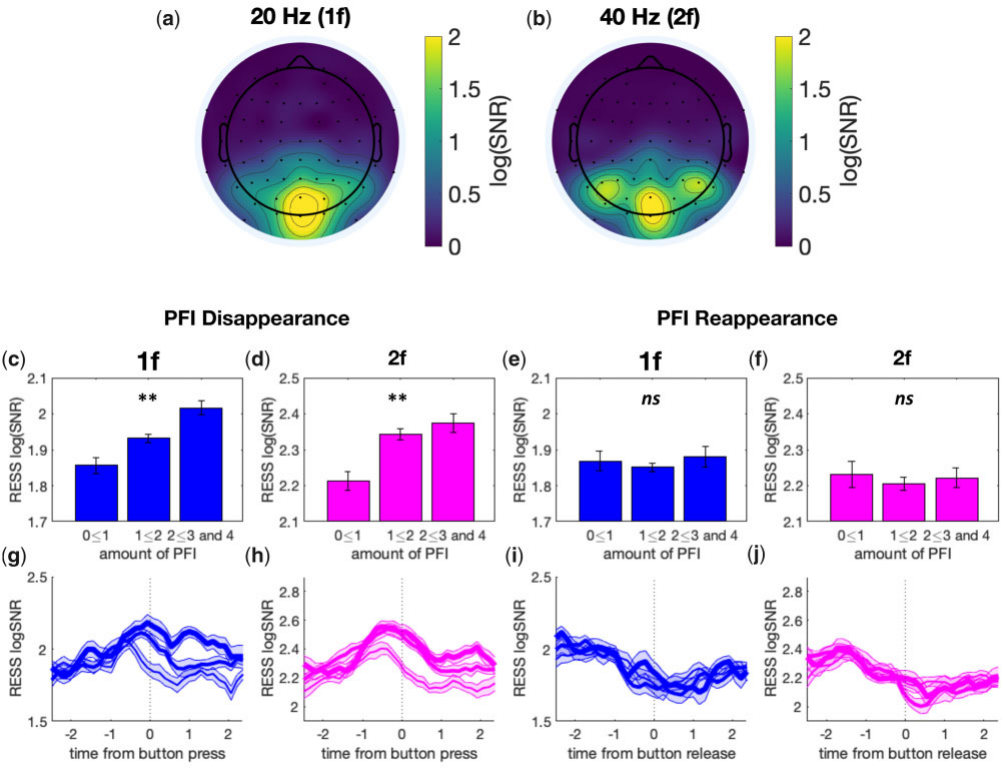
The amount of PFI is correlated with the RESS log(SNR) around PFI disappearances, but not reappearances. (**a, b**) Topoplots for the mean log(SNR) at 20 Hz (1f; stimulus flicker), and 40 Hz (2f; stimulus harmonic) for background-related SSVEPs. The mean is taken across participants over all epochs, excluding PMD. (**c–f**) Bar graphs for the mean RESS log(SNR) over −2.5 to 2.5 seconds as a function of the amount of PFI. (**g–j**) The time course of RESS log(SNR) around the button press or release, separated by the amount of PFI, with three levels indicated by the thin, middle and thick lines. Error bars and shading indicate 1 SEM across participants (adjusted for within-participant subject comparisons; Cousineau 2005).

We investigated whether the amount of PFI reported would reflect changes in RESS log(SNR) across participants. For PFI disappearances and reappearances, we averaged this over [0, +3] seconds and [−3, 0] seconds with respect to the button press or release, respectively. We call this sum of the number of buttons pressed over these time periods ‘the amount of PFI’. As the frequency of PFI events varied greatly between participants (Supplementary Fig. 2), we performed an event-by-event analysis (Fujiwara et al., 2017) before across-participant averaging. For this analysis, all PFI events were epoched around button press/release, and sorted in descending order based on the amount of PFI. We then resampled each participants PFI events along the trial-dimension to 100 trials, in order to normalize the event count per participant. On this normalized event count, we then grouped all events when the amount of PFI was between 0 and 1, 1 and 2, or greater than 2 (predetermined by button-press). The average RESS log(SNR) during these periods, categorized by the amount of PFI reported, is shown in Fig. 4. Additional methods and a schematic pipeline for this entire procedure are displayed in Supplementary Material and Supplementary Fig. 2.

### Reconstruction analysis to compare the impact of multiple target disappearances and reappearances on SNR, during PFI and PMD

**Figure 5.**
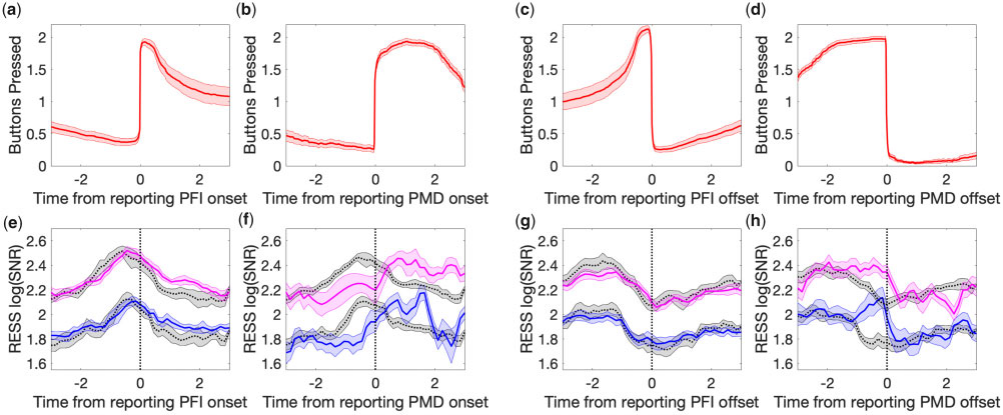
Reconstruction analysis. (**a–d**) display mean button-press during target disappearance and reappearance, (**e–h**) show the observed RESS log(SNR) time course across participants during these periods (1f, blue; 2f, magenta), as well as reconstructed data (grey). The observed SNR time course is shown from test trials which were not used to construct the reconstruction kernels. The correlation (*R*^2^) between the observed SNR and the predicted SNR was used to quantify prediction accuracy. Note that for all panels, time 0 is always defined by a button press (a, b, e, f) or release (c, d, g, h). Shading represents 1 SEM across participants (corrected for within participant comparisons; Cousineau 2005).

We included PMD to compare with PFI, yet the temporal profile of these phenomena differs markedly. This is because PFI can accumulate at multiple targets within close temporal proximity, yet PMD were programmed to always occur simultaneously. To quantify the similarity between PFI and PMD while accounting for these differences in temporal structure, we performed a reconstruction analysis. Specifically, we estimated whether changes to log(SNR) during PMD could be accurately modelled using the recorded changes in log(SNR) from PFI periods. For this analysis, we averaged the response to PFI using 75% of trials per participant (excluding PMD), and retained this temporal change in log(SNR) as a reconstruction kernel. Using this kernel, we then reconstructed the expected temporal profile of log(SNR) for the remaining 25% of trials, based on the timing of button-press responses per participant. The comparison between the reconstructed (expected) changes based on kernels, and actual log(SNR) during PFI and PMD in this remaining 25% of trials are displayed in Fig. 5. A more detailed description and schematic of this procedure are contained in the Supplementary Information and Supplementary Fig. 3.

### Analysis of SNR timing differences

Past research on binocular rivalry has indicated that perceptual alternations between frequency-tagged stimuli are captured in the time course of SSVEP-SNR (e.g. Brown and Norcia 1997; Tononi *et al.*, 1998; Cosmelli *et al.* 2004; Zhang *et al.*, 2011; Katyal *et al.*, 2016). We were interested to see whether changes in SNR could also predict button presses/releases in our multi-target PFI paradigm, and compared the onset at which 1f and 2f responses indicated a change in perception. For background related SNR, this requires overlaying the same SSVEP response (e.g. 20 Hz) during both disappearance and reappearance, as opposed to comparing two unique simultaneous SSVEPs as is usually done in binocular rivalry research (e.g. Zhang *et al.* 2011; Katyal *et al.* 2016).

We compared the disappearance and reappearance time courses using running paired samples *t*-tests (two-tailed) at each time point. Clusters of significant time points were identified which satisfied *P* < 0.05 (uncorrected) over a minimum of two time points (or 300 ms) in our moving-window SNR. Per participant, the first time point in these clusters was taken as when the SNR differentiates between target disappearance and reappearance in our analysis. We then compared the earliest time-points detected by this procedure for 1f and 2f, using a two-tailed paired-samples *t*-test. We also performed the same analysis to compare the time course of SNR during PMD. Note that this cluster-procedure is distinct from the procedure described below, which retains the largest cluster for non-parametric tests.

### Spatial correlation analysis

To perform the spatial correlation analysis, we calculated the time-course of a 64 channel correlation between 1f and 2f log(SNR). Due to differences in the number of PFI and PMD, we downsampled (with replacement) the number of PFI events to 24, which was the maximum number of PMD available. We then calculated the correlation between 1f and 2f, separately within PFI and PMD for this subset of trials, and repeated this analysis 100 times to obtain a distribution of downsampled correlation values. The mean correlation value from this downsampled distribution was then used to compare the spatial correlation of PFI and PMD at each time point around button press and release. The results of this analysis are displayed in Fig. 7.

### Statistical analysis—EEG

All statistical analyses were performed using Matlab (ver: R2016b) and jamovi (ver 0.9; The jamovi project, 2018). To assess the significance of SSVEP peaks in the EEG spectra, we corrected for multiple comparisons with a false discovery rate (FDR) of 0.05 (Benjamini and Yekutieli 2001; Benjamini *et al.* 2006). For corrections of multiple comparisons on the time courses, we used temporal cluster-based corrections (Maris and Oostenveld 2007). For this analysis, the sum of observed test-statistics (e.g. *t* scores) in a temporally contiguous cluster were retained for comparison with a permutation-based null distribution. Specifically, first, we detected any temporally contiguous cluster by defining a significant time point as *P* < 0.05 uncorrected (Maris and Oostenveld 2007). Then, we concatenated the contiguous temporal time points with *P* < 0.05 and obtained a summed cluster-level test statistic for the cluster. Second, we repeated this procedure after shuffling the subject specific averages within each participant 2000 times. From each of the 2000 shuffled data, we obtained the summed cluster-level test statistics at contiguous temporal time points with *P* < 0.05 uncorrected, which served as a null distribution. We regarded the original observed effect to be significant if the original summed cluster-level statistics exceeded the top 97.5% of the null distribution of the summed statistics (as *p*_cluster_ < 0.025).

## Results

### Behavioural analysis: a synergistic effect of multi-target PFI

First, we examined behavioural data to investigate whether our unique multi-target design had captured an interaction between the four simultaneously presented peripheral targets. Previous research has suggested that neighbouring targets within a single visual quadrant may disappear together (De Weerd *et al.* 1998). Our design allowed us to examine whether much more widely distributed peripheral targets also interact. Such an interaction would be non-trivial if occurring across the disparate retinotopic locations of all four quadrants of the visual periphery, and would imply the involvement of potentially high-level neural mechanisms that have access to these long-range relations (Wagemans *et al.* 2012).

While simultaneous disappearances of 3 and 4 targets were rare (only 2.9 events per trial; blue bars Fig. 3a), when they happened, the event tended to be sustained for a long duration (2.2 seconds, Fig. 3b). As a result, the total duration of 3 and 4 target invisibility (7.6 seconds per trial, Fig. 3c) is comparable to that of 2 target invisibility and longer than that of 1 target invisibility, which happened at the highest rate (8.8 events per trial, 5.8 seconds in total per trial). We formally tested this linear trend by linear mixed effects analysis and likelihood ratio tests (with random intercepts for each subject). We performed likelihood ratio tests between the full model and a restricted model which excluded the factor of interest (Glover and Dixon 2004; Winter 2013). The number of invisible targets (nPFI) significantly affected (i) the number of PFI events per trial (χ^2^(2) = 47.83, *P *=* *4.1 × 10^−11^), (ii) the average duration of PFI per event (χ^2^(2) = 23.59, *P *=* *7.53 × 10^−6^) and (iii) total PFI duration per trial (χ^2^(2) = 7.27, *P* = 0.026).

These significant trends imply that interactions among distant targets occur in a synergistic way, and that when one target is invisible it is often accompanied by other invisible targets. To directly test if this is the case, or if these trends occur by chance, we employed a shuffling analysis (see Materials and methods). In the shuffled data, the number of PFI events per trial decreased as the number of invisible targets (nPFI) increased, which is similar to what we observed in the empirical data (10.8, 7.1 and 2.7 events per trial for 1, 2 and 3, and 4 target invisibility; Fig. 3a, grey bars). However, the trend for shuffled data was quite different for the average durations per PFI event, which were roughly equal across nPFI in shuffled data (2, 1.6 and 1.3 seconds, respectively; Fig. 3b), and the total duration of PFI per trial, which decreased as a function of the number of invisible targets (16.6, 9.7 and 3.8 seconds, respectively; Fig. 3c).

To statistically evaluate these trends between the observed and the shuffled data, we compared the slopes of the linear fit for each of the three PFI variables as a function of the number of invisible targets (nPFI; 1, 2, 3 and 4). For this analysis, we fit a linear model (first-order polynomial) to the observed data across participants, and retained the slope (β) as our observed test statistic. We also fit the same linear model to each of *n *=* *1000 sets of shuffled data, computed from the shuffled trials across participants. For all variables, the observed slope was outside the top 97.5% of the slopes in the shuffled data (corresponding to two-tailed *P* < 0.05, Fig. 3d–f). Notably, the observed positive slope for PFI duration and total duration in Fig. 3b and c is contrary to the expected negative slope produced by our shuffled data. This negative slope in shuffled data implies that if there are no spatial interactions between distant targets, we should expect the simultaneous invisibility of 3 and 4 targets to be unlikely, and sustained for a shorter duration. By contrast, as more targets are involved in PFI, the longer the disappearances are sustained, strongly suggesting a facilitative interaction between invisible peripheral targets. We return to this synergistic effect of multi-target PFI in our Discussion.

### SSVEP time course reveals graded changes in conscious perception

Next, we turned to our EEG analysis of SSVEPs. Both 1f (20 Hz) and 2f (40 Hz) frequency-tagged responses to our background display were strongest at POz (on average, across participants). However, the spatial topography differed between 1f and 2f (Fig. 4a and b). The 1f response was localized to midline occipital electrodes, while the 2f response extended beyond these regions to include lateral parieto-occipital and parietal electrodes. Due to the variance across participants in the spatial distribution of these responses, we employed RESS (Cohen and Gulbinaite 2016), to optimally extract the SNR per participant, and to avoid multiple comparisons across electrodes (see Materials and methods). Using RESS, we analysed the time-course of log(SNR) for background-related 1f and 2f responses. All log(SNR) values we present are the RESS log(SNR), except for the spatial correlations presented in Fig. 7.

A second qualitative insight concerned the time-course of log(SNR) activity: 1f and 2f increase just before button press when targets disappear (at time = 0), and increase with the amount of PFI (Fig. 4c, d, g, h). Similarly, log(SNR) for 1f and 2f decrease just before button release at target reappearance, but there is no dependence on the amount of PFI (Fig. 4e, f, i, j). To quantitatively compare these differences, we split SNR time courses based on the amount of PFI. Figure 4c–f show the mean log(SNR) over each 6 second epoch, separately averaged for events with the amount of PFI between 0 and 1, 1 and 2 or greater than 2. Around the target disappearance events, we found a significant linear effect for the amount of PFI on the SNR for both 1f (χ^2^(1) = 8.75, *P* = 0.003) and 2f (χ^2^(1) = 8.21, *P* = 0.004) responses to background flicker (Fig. 4c and d). Around target reappearance events, by contrast, the amount of PFI did not significantly affect the SNR (Fig. 4e and f, 1f; *P* = 0.76; 2f; *P* = 0.83). Figure 4g–j displays the time course of the SNR separately for each level of the amount of PFI.

### Reconstruction analysis: SNR time courses during PFI are distinct from those during PMD

The previous analysis has shown that changes to the log(SNR) of background flicker were related to the amount of PFI, we next considered whether these changes could be distinguished from the changes evoked by PMD. Contrasting PFI and PMD could isolate the neural substrates which are unique to an endogenous change in perception (as in PFI), as opposed to a physically induced change (as in PMD). To account for whether the changes in log(SNR) during PFI are distinct from those during PMD, we performed an SNR-reconstruction analysis. In brief, we used 75% of trials to estimate reconstruction kernels, which were the changes to log(SNR) during PFI in this ‘training’ data. We then applied these kernels to the remaining 25% of ‘test’ trials, aligning each kernel to the recorded button press time-points (see Materials and methods and Supplementary Fig. 3). We then compared the predicted time course of log(SNR) based on the reconstruction kernels, with the actual time course in the test trials during genuine PFI and PMD. Figure 5 visualizes the high quality of prediction for the genuine PFI (Fig. 5e and g) and the poor predictive quality for PMD (Fig. 5f and h). These differences in predictive accuracy of our SNR reconstruction analysis implicate distinct neural substrates for PMD and PFI.

We quantified reconstruction prediction accuracy as the degree of correlation between the predicted and the observed time course. Specifically, we calculated *R*^2^ between the respective epoched log(SNR) around button press/release events during genuine PFI and PMD. For both 1f and 2f, the predicted SNR was correlated more strongly with genuine PFI than the PMD, for both disappearances and reappearances (Table 1). Using three-way repeated measures analysis of variance (ANOVA) (Table 2), we confirmed that the prediction accuracy is significantly better for the genuine PFI than PMD (main effect: *F*(1, 21) = 151.01, *P *=* *4.7 × 10^−12^). We found no or weak main effects and interactions due to other factors (i.e. 1f vs. 2f, disappearances vs. reappearances).

**Table 1.** Prediction accuracy (as *R*^2^) across reconstruction analyses

	PFI 1f disap.	PFI 2f disap.	PMD 1f disap.	PMD 2f disap.	PFI 1f reap.	PFI 2f reap	PMD 1f reap.	PMD 2f reap.
Mean	0.50	0.54	0.08	0.13	0.45	0.49	0.12	0.13
Std. error mean	0.05	0.05	0.03	0.03	0.05	0.05	0.03	0.04
Standard deviation	0.23	0.22	0.12	0.14	0.25	0.22	0.16	0.17

**Table 2.** Results of 2 × 2 × 2 repeated measures ANOVA on *R*^2^ values

	Sum of squares	df	Mean square	*F*	*P*	Partial η²
PFI vs. PMD	6.24	1	6.24	151.01	<0.001	0.88
Residual	0.87	21	0.04			
1f vs. 2f	0.06	1	0.06	0.94	0.342	0.04
Residual	1.25	21	0.06			
Disap. vs. Reapp.	0.01	1	0.01	0.45	0.512	0.02
Residual	0.67	21	0.03			
(PFI vs. PMD) × (1f vs. 2f)	0.00	1	0.00	0.07	0.792	0.00
Residual	0.65	21	0.03			
(PFI vs. PMD) × (Disap. vs. Reapp.)	0.05	1	0.05	5.34	0.031	0.20
Residual	0.21	21	0.01			
(1f vs. 2f) × (Disap. vs. Reapp.)	0.01	1	0.01	0.47	0.499	0.02
Residual	0.39	21	0.02			
(PFI vs. PMD) × (1f vs. 2f) × (Disap. vs. Reapp.)	0.00	1	0.00	0.28	0.603	0.01
Residual	0.34	21	0.02			

*Note*: Type 3 sums of squares.

### Timing differences: 1f and 2f background-related responses are temporally distinct during PFI

Even though our reconstruction analysis predicted both the 1f and 2f components of background-related SNR during PFI events, these harmonic responses were topographically distinct (Fig. 4a and b). As there is a nascent literature suggesting that SSVEP harmonics may correspond to separate cortical loci (e.g. Pastor *et al.* 2007), and cognitive processes (Kim *et al.* 2011), we next investigated these spatiotemporal differences in more detail.

**Figure 6.**
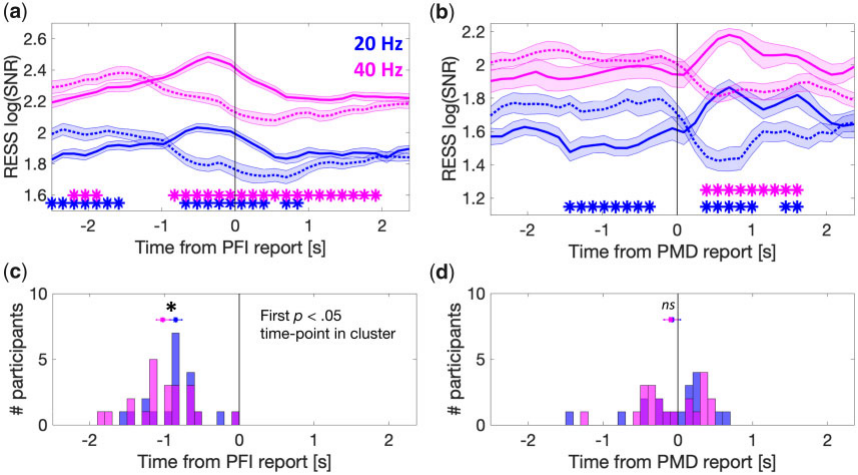
Distinct temporal profile of the harmonic responses. (**a, b**) Group-level relative time course of the 1f (20 Hz, blue) and 2f (40 Hz, magenta) RESS log(SNR) during PFI events (a) and PMD (b). Solid and broken lines represent disappearance and reappearance, respectively. (**c, d**) Participant-level histograms for the first significant time point when comparing between the RESS log(SNR) for disappearance and reappearance during PFI (c) and PMD (d). Horizontal lines indicate 1 SE about the mean corrected for within-subject comparisons (Cousineau 2005).

First, we superimposed the time courses for disappearances/reappearances in the same plot, and calculated the periods at which the log(SNR) significantly differed between them. For 1f (Fig. 6a and b, blue), the log(SNR) during disappearances (solid lines) became larger than that during reappearances (dotted lines), consistent with an increase in background-SNR during the phenomenal experience of targets becoming filled-in by the background. This effect occurred from 0.67 seconds prior to subjective report (paired *t*-tests, *p*_cluster_ < 0.001). Notably, these effects occurred 1.06 seconds later for PMD (Fig. 6b, from 0.39 seconds after the subjective report, *p*_cluster_ < 0.001). For 2f (Fig. 6a and b, magenta), the log(SNR) also became larger during disappearances than reappearances from 0.97 seconds prior to report (*p*_cluster_ < 0.001), and again, were shifted roughly 1.36 seconds compared to the PMD-related time course (Fig. 6b, from 0.39 seconds after the subjective report; *p*_cluster_ < 0.001).

The observed divergence in the time for significant changes to 1f and 2f SNR seemed quite large (0.3 seconds) given that both 1f and 2f were evoked from the same stimulus, using identical participants and events. As such we performed an exploratory analysis, and further investigated if this effect could be observed at the participant level. For this analysis, we calculated for each participant the first time point at which the strength of background log(SNR) during disappearance was significantly greater than during reappearance, using running paired *t*-tests. The first time-point in a temporally contiguous cluster was retained per participant for both 1f and 2f log(SNR), and then compared at the group level. Using this criterion, we found that during PFI, 2f responses significantly differed at −1.02 seconds (SD = 0.41), 170 ms earlier than 1f responses, at −0.85 seconds (SD = 0.37, Wilcoxon signed rank test, z = 2.13, *P* = 0.012). No difference was observed in the timing for the PMD-related 1f and 2f time courses (*P* = 0.14). These findings were confirmed using a jack-knife procedure (see Supplementary Materials). The distribution of participant-level first significant time-points are displayed in Fig. 6c and d.

### Spatial correlation: 1f and 2f background responses are spatially distinct during PFI

Figure 6 demonstrated that the temporal dynamics of 1f and 2f differed around PFI. These SNR time-series, however, are dependent on the fit of RESS spatial filters, and the spatial distribution of frequency-tagged responses is also known to vary with changes in perception (Tononi *et al.* 1998; Srinivasan 2004; Srinivasan *et al.* 2006, 1999). To test this possibility, we continued our exploratory analysis and performed a spatial correlation analysis, to examine the topographic distribution of 1f and 2f responses during PFI in 64-channel log(SNR) data (see Materials and methods).

**Figure 7.**
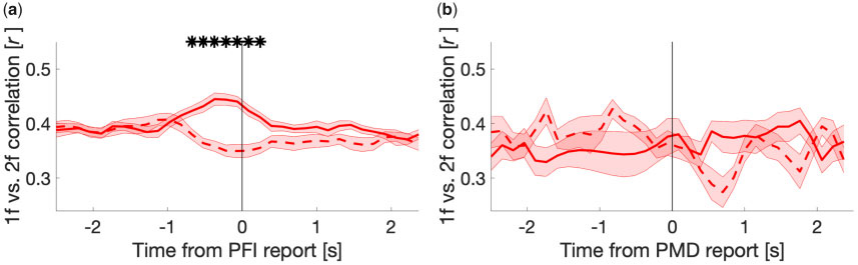
Time course of the spatial correlation coefficient (*r*) between 1f and 2f (non-RESS) log(SNR) across 64 electrodes. Correlation coefficient was computed across 64 electrodes at each time point per participant. The mean time courses of correlation coefficients are shown for target disappearance (solid) and reappearance (dotted) around (**a**) PFI and (**b**) PMD. For PFI, we show the mean correlation value obtained after downsampling PFI events to 24 (the maximum number of PMD), over 100 repetitions of this downsampling procedure. Asterisks denote the time points with significantly different correlation coefficients between PFI disappearances vs. reappearances (paired t-tests, cluster corrected). Shading reflects the SEM across participants corrected for within-subject comparisons (Cousineau 2005).

When targets disappeared during PFI, the 64-channel spatial correlation between 1f and 2f transiently increased (Fig. 7a). The difference between the time courses was significant for the time-window −0.67 to 0.25 seconds around subjective report (paired *t*-tests at each time point, *p*_cluster_ < 0.001). In contrast, when targets were removed during PMD, the spatial correlation between harmonics remained constant (Fig. 7b). The same pattern of results was maintained when using a parietal or occipital sub-region of electrodes (but no change in correlation was seen for frontal or temporal electrodes), indicating that synchronous changes in predominantly parieto-occipital SNR were responsible for changes to the whole-head correlation over time (shown in Supplementary Fig. 6). The same pattern was also observed when subtracting the mean log(SNR) per channel prior to calculating this spatial correlation over time, confirming this change in correlation was driven by a change in the spatial extent of SNR, rather than transient increase in SNR. These results support a distinction between 1f and 2f responses that is unique to PFI and suggest that the timing differences observed (Fig. 6) may be partially driven by changes to the spatial distributions of 1f and 2f responses.

## Discussion

We combined a multi-target PFI paradigm with frequency-tagged EEG. This combination has revealed that changes to the contents of perception can be tracked behaviourally across multiple simultaneous locations, and that gradual changes to neural markers accompany these reports in the frequency-tagged EEG. We also found an unexpected asymmetry for graded disappearances and reappearances (Fig. 4), and spatiotemporal distinctions between steady-state visual evoked potential (SSVEP) harmonics (1f and 2f background responses, Figs 6 and 7). Here, we discuss these findings focusing on several advantages of our experimental paradigm for future NCC research.

### Multi-target PFI to track changes in conscious perception

Frequency-tagging has been used to study the NCC, mainly in combination with binocular rivalry (Brown and Norcia 1997; Tononi *et al.* 1998; Sutoyo and Srinivasan 2009; Zhang *et al.* 2011; Jamison *et al.* 2015; Katyal *et al.* 2016). Unlike stimuli normally used in binocular rivalry, where changes in perception are often patchwork or complex (Wilson *et al.* 2001; Knapen *et al.* 2011); but also see (Blake *et al.* 1992; O’Shea *et al.* 1997), perceptual changes during PFI are complete and near instantaneous, suggesting PFI can prove to be a useful psychophysical tool to study the NCC. In our multi-target and multi-response task, participants report on graded perceptual changes with each button, which also indicate where in space these changes occur. Our simultaneous EEG enabled the amount of change in the contents of consciousness to be recorded (i.e. the amount of PFI), and in principle, which targets disappear can also be addressed in future research.

This approach revealed an asymmetry between the neural correlates of disappearances and reappearances: background SNR increased with the amount of PFI at disappearance, but not reappearance. One possible explanation is a difference in the spatial and temporal predictability of these events. Increased spatial predictability follows from the fact that reappearances can only occur at locations where a target has already disappeared moments earlier. Increased temporal predictability follows from the fact that the duration of PFI are relatively short compared to periods of visibility (Fig. 3). In support of this difference during PFI periods, differences in predictability have also been measured via greater phasic pupil responses to target disappearances than reappearances during motion-induced blindness (Kloosterman *et al.* 2015; Thomas *et al.* 2017), a paradigm that may be closely related to PFI (Hsu *et al.* 2004, 2006; New and Scholl 2008; Devyatko *et al.* 2017). Another possibility is that during PFI, microsaccades may have shifted the position of targets on the retinae, which is known to counteract filling-in (Martinez-Conde *et al.* 2006). While we did not control for microsaccades in our design, a reappearance driven by eye-movements would also be consistent with the asymmetry we have observed, and can be tested in future experiments with eye-tracking hardware.

### A synergistic effect demonstrates the spatial-range of PFI mechanisms

Our results are relevant to two popular models of PFI. The first model proposes the primary substrate of PFI are neurons in *early retinotopic areas* corresponding to target regions, which are activated via neurons corresponding to their surrounds through lateral connections (De Weerd *et al.* 1995; Pessoa *et al.* 1998). The low-level model specifically proposes that the mechanisms of PFI are confined to retinotopic cortex, with disappearance happening via a two-stage process. A first stage of seconds-long target-boundary adaptation is followed by a second stage of near instantaneous interpolation of the target location by surrounding visual features (Spillmann and De Weerd 2003). The second is a high-level symbolic model, whereby filling-in occurs when the visual system ignores an absence of information (Kingdom and Moulden 1988; Dennett 1991; O’regan 1992). In this model, the phenomenon of filling-in is realized at a higher representational level, and not as the result of any point-to-point representation in early visual cortex. Instead, a region devoid of information is symbolically labelled as ‘more of the same’ background, and thus is rendered invisible.

In favour of the low-level model, previous electrophysiological data have shown increased spike rates in regions responding to a filled-in pattern in monkeys (De Weerd *et al.* 1995). Importantly, De Weerd *et al.*’s (1995) single-unit study did not obtain subjective reports from their non-human primates. With human participants, Weil *et al.* (2007) recorded decreases in average SSVEP power for targets during PFI periods, yet did not provide the time-course of response-locked frequency-tagged activity. A critical aspect of our novel approach, missing from these earlier studies, is our response-locked analysis which allowed us to examine if the exact timing of increases in neural activity precedes or follows the onset of a perceptual disappearance. By recording simultaneous subjective reports, we conclude that an increase in background SNR precedes PFI events. This slow, seconds-long increase in background-related SNR prior to PFI events supports an active mechanism as a catalyst for PFI, which is central to the low-level model (but also consistent with some high-level explanations). Our SNR reconstruction analysis also showed significant differences between PFI and PMD, suggesting that the presence of competitive mechanisms supporting perceptual disappearances in PFI are an important contribution to these background-related SNR differences. The potential involvement of neural responses from a visual background region onto surrounded visual space is also supported by a recent finding in individuals with Charles-Bonnet Syndrome (CBS), who perceive visual hallucinations in response to vision loss within visually deafferented space. It was shown that the visual periphery that surrounds these visually deafferented regions shows enhanced activation in individuals with CBS, compared to controls (Painter *et al.* 2018).

On the other hand, the low-level model is difficult to reconcile with our behavioural findings, which more readily support that filling-in happens in higher-level visual areas, in accordance with symbolic models. Specifically, we observed a synergistic effect among spatially distant targets, wherein targets from separate retinotopic locations interacted to remain invisible for longer during PFI. This interaction implies the involvement of neurons that have larger receptive fields, typically found only in higher-level visual areas (Yoshor *et al.* 2007; Dumoulin and Wandell 2008). This across-quadrant facilitatory interaction contrasts with the results of de Weerd *et al.* (1998) which were in support of the low-level model. There, de Weerd *et al.* (1998) argued that differences in PFI onset times for separate locations indicated that PFI mechanisms operate with a limited spatial range. Instead, the synergistic effects we have observed may point to a mechanism that facilitates perceptual grouping (Wagemans *et al.* 2012). This strongly argues against the limited spatial range of PFI mechanisms, implying the involvement of higher-cortical regions that can influence neural activity across visual hemifields.

Grouping may also interact with attentional mechanisms. Indeed, attending to shared features, such as temporal modulation, has been shown to enhance the binding of distributed visual regions into a perceptual group (Alais *et al.* 1998). As attending to shared features such as colour (Lou 1999) or shape (De Weerd *et al.* 2006) also increases the disappearance of peripherally presented targets, fluctuations in attention to the targets as a group may also have impacted on multiple-locations synergistically. Alternatively, the simultaneous disappearance of multiple targets could be due to random fluctuations of the brain’s response to the background (potentially also modulated by attention). Since the background surrounds all targets, a temporary increase in response could affect the visibility of all targets simultaneously.

Overall, our results show that PFI does not result purely due to local adaptation processes in retinotopic cortex, and extends our understanding of the spatial range of PFI mechanisms. Cortical regions with larger receptive fields may account for this synergistic effect of enhanced PFI, a hypothesis that may be tested in future research with source localization techniques.

### Spatiotemporal profiles of 1f and 2f background SSVEP are distinct

Another insight that arose from our application of SSVEP to study PFI regards the difference in spatiotemporal profiles of 1f and 2f responses (Figs 6 and 7). As we used a single-taper method, with a fixed time-window and step-size, this latency difference cannot be due to differences in temporal smoothing between harmonics (for the theory and simulation of these smoothing effects in time and frequency domains, see Supplementary Material of Takaura et al., 2016). This difference was specifically modulated around the time of PFI. In the literature, 1f and 2f are traditionally considered to be similar, as they are dictated by the same stimulus input (Norcia *et al.* 2015). Recently, this assumption has been challenged by finding that 2f responses are more sensitive to attentional modulation under some conditions, such as when symmetrically oscillating light-dark flicker (Pei *et al.* 2002; Kim *et al.* 2007; Kim and Verghese 2012), and differ in their topography, with an increased lateralization of 2f responses (Appelbaum *et al.* 2008; Kim *et al.* 2011), involving parieto-occipital cortex (Pastor *et al.* 2007). While we also observe an increased spatial distribution of 2f—as 1f was strongest over mid-occipital sites and 2f extended laterally (Fig. 4)—the flicker stimuli used in our experiments differ from those studies that optimized differentiating 1f from 2f (e.g. Kim *et al.* 2011). As such, extending this interpretation to our findings should be done with caution, but the temporal advantage of 2f compared to 1f prior to PFI would be consistent with a covert attentional modulation of 2f that instigates a perceptual change. Future studies with an explicit attentional manipulation will be needed to confirm whether the harmonic differences we have reported are due to the allocation of attention.

## Conclusions

Here we have extended efforts to refine NCC paradigms, by combining PFI with frequency-tagging. With our novel flickering background display, we successfully induced the PFI of multiple targets, which participants were able to accurately report on without much training. We were able to mimic the phenomenological dynamics of PFI with our physical PMD. Although we did not formally test the discriminability of PMD compared to PFI, no participants spontaneously reported their awareness of PMD. Despite the similarity between PFI and PMD, we revealed significant differences in their respective neural substrates through our SNR reconstruction analysis, which suggest that these differences are due to the presence of competitive mechanisms supporting perceptual—but not physical disappearances. While the current study focused on tagging the background, future studies may also reveal the exact nature of such competitive mechanisms by simultaneously tagging both the targets and background during PFI. Unexpectedly, we found significant differences in the effect that the amount of PFI had on SNR during disappearances and reappearances. While we suggest that the difference could be due to differences in the level of predictability, to address this question more thoroughly, future studies could manipulate the disappearance frequency of targets by manipulating their stimulus properties (e.g. contrast or eccentricity or by changing the number of targets to be reported. We hope that our approach of combining the under-utilized PFI paradigm and SSVEP techniques will refine efforts to establish the NCC, and various novel designs to address other central questions in cognitive neuroscience, such as the neural basis of attention and consciousness (Tsuchiya and Van Boxtel 2013; van Boxtel and Tsuchiya 2015), and establishment of no-report paradigms (Tsuchiya *et al.* 2015).

## Data availability

All raw data, code reproduce the analysis and code to re-run the experiment are located at https://osf.io/uwxeh/. 

## Supplementary Material

niaa002_Supplementary_Data
