# Supplementary material for: A multiple-response frequency-tagging paradigm measures graded changes in consciousness during perceptual filling-in
Source: Neurosci Conscious. 2020 Apr 12;2020(1):niaa002. doi: 10.1093/nc/niaa002 (PMC7151726; doi:10.1093/nc/niaa002)
Supplement: niaa002_Supplementary_Data [file niaa002_supplementary_data.docx]

# Supplementary Materials

#

# Supplementary Methods:

***PMD analysis during reappearance***

In addition to the button press responses to PMD onset (Figure 2), we also analyzed button release following target return at the end of PMD periods. This analysis progressed through the same sub-sampling and bootstrapping procedure as described in ***Participant and trial exclusion based on PMD****,* with one exception. Rather than calculate the first time point at which the observed data exceeded the CI of our null-distribution, we used the first time point at which the observed data fell *within* the CI of our null-distribution as the reaction-time of interest. We interpret this reaction time as the first time after PMD offset, at which the observed button-press likelihood returned to the baseline button-press likelihood. This difference is illustrated in Supplementary Figure 1a, for a single participant.

Repeating our criteria for participant rejection, only a single participant’s time-course failed to return within the baseline button-press likelihood two seconds after PMD offset. This participant was one of the four already excluded for failing to experience PFI during most of the experimental session, so no further action was taken. We did not exclude individual trials based on the likelihood of button release following PMD offset because we did not want to exclude any trial which included genuine PFI that immediately followed PMD offset.

####

####
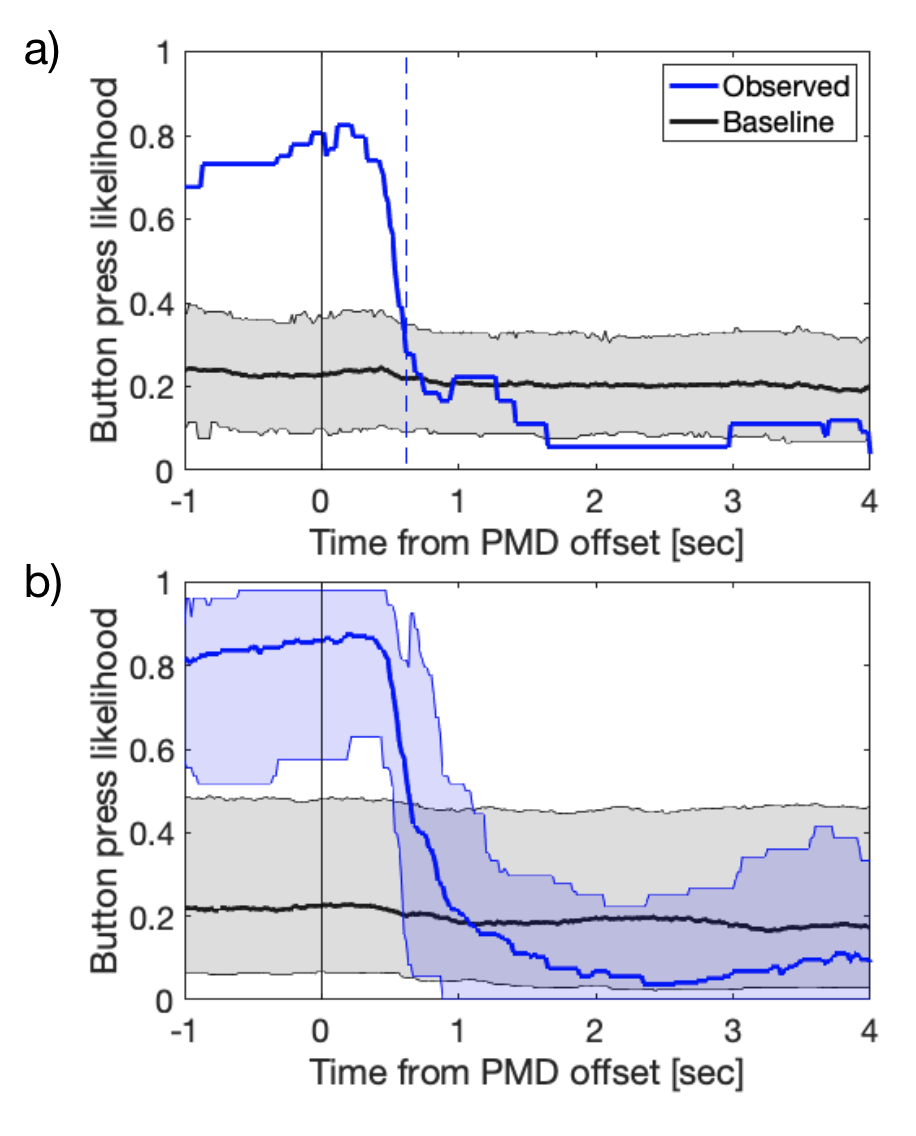


#### Supplementary Figure 1. PMD analysis following the physical return of flickering targets at PMD offset. a-b) display the likelihood of button-press time-courses for observed (blue) and bootstrapped data (grey). a) Example response for a single participant when targets return at PMD offset. The first time point that the observed likelihood of button press (blue) fell within the bootstrapped CI (grey) corresponds to the reaction time (0.62 second for this participant, marked with a vertical dashed blue line). b) The mean time course for the likelihood of button press and its bootstrapped sets across participants, shown in blue and grey respectively. Shading represent the CI (computed with logit transform and presented after reverse transform) across participants.

## EEG acquisition and preprocessing

Throughout each session whole-head EEG was recorded with 64 active electrodes arranged across an elastic-cap (Brain Products, ActiCap) according to the international 10-10 system. Electrode impedances were kept below 10 kΩ prior to experimentation, and recorded using the default reference (FCz) and ground electrode (AFz) via Brainvision recorder software (sampling rate = 1000 Hz, offline bandpass of 0.5-70 Hz). All EEG data was stored for offline analysis using custom MATLAB scripts (Ver: R2016b), as well as the EEGLAB [(Delorme & Makeig, 2004)](https://paperpile.com/c/ROJwbu/F8Pi) and Chronux [(Bokil, Andrews, Kulkarni, Mehta, & Mitra, 2010)](https://paperpile.com/c/ROJwbu/8bU2) toolboxes. All EEG channels were first re-referenced to the average of all electrodes at each sample and downsampled to 250 Hz. We further applied a Laplacian transform to improve spatial selectivity of the data, which is known to contribute minimal contamination to the SSVEP when using rhythmic-entrainment source separation (RESS; [Cohen & Gulbinaite, 2016)](https://paperpile.com/c/ROJwbu/8BHE), which we used to extract SSVEP responses. RESS creates a map of spatial weights to optimally extract SSVEP signal, without relying on a priori or post-hoc electrode selection.

## SSVEP analysis via rhythmic entrainment source separation (RESS).

In standard SSVEP analysis, the SNR is examined by averaging across electrodes within a region of interest or selecting one electrode in a certain way (e.g., prior hypothesis, anatomical localization or separate datasets). An alternative to this classic approach is RESS, which creates a map of spatial weights across all electrodes which optimize the SNR at a particular frequency, tailored for each participant. Specifically, RESS functions by creating linear spatial filters to maximally differentiate the covariance between a signal flicker frequency and neighbourhood frequencies, thereby increasing the signal-to-noise ratio at the flicker frequency. After obtaining signal and neighbourhood covariance matrices, the eigenvector with the largest eigenvalue is used as channel weights to reduce the dimensionality of multi-channel data into a single component time course, which reduces multiple comparisons across channels in statistical testing.

After epoching all data using the time-windows -3000 to -100 ms and 100 ms to 3000 ms peri button press/release, we then constructed RESS spatial filters per participant, avoiding PMD. We constructed RESS spatial filters from 64-channel EEG, by extracting signal data following a narrow-band filter via frequency-domain Gaussian, centered at flicker frequencies (20 and 40 Hz, full-width at half maximum = 1 Hz). We proceeded by selecting broadband (unfiltered) neural activity to construct reference covariance matrices. We selected broadband EEG as our reference after confirming that improvements to the SNR at both the 20 and 40 Hz signal were statistically equivalent. Comparing signal to broadband activity as a reference, as opposed to immediate neighbourhood frequencies, has previously been shown to allow the optimization of SSVEP signals using RESS [(Cohen & Gulbinaite, 2016)](https://paperpile.com/c/ROJwbu/8BHE).

Critically, we performed the above procedure without distinguishing whether targets were disappearing or reappearing in order to reduce the possibility of overfitting. If we were to construct separate filters for periods around the time of target disappearance and reappearance, then any differences between these conditions could be due to differences in the obtained filters, or overfitting of the filters prior to our condition comparisons. After application of the RESS spatial filters, we calculated the time course of SSVEP log(SNR) from the RESS component time courses, separately for each background flicker of interest as described above (*SSVEP Signal-to-Noise Ratio (SNR) calculation*). The main results of our analysis also hold with more conventional SSVEP analysis, such as when focusing on only parieto-occipital electrodes.

## SNR time-course data cleaning

Preliminary analyses revealed a sharp and consistent decrease in 40 Hz log(SNR) amplitude which was time-locked to the beginning of each PMD. Subsequent inspection of recorded screen flip-times revealed a lag in background stimulus presentation (16.7-33.3 ms duration) at PMD onset, which resulted in the background pixels for one presentation frame being skipped. This caused an artifact in the spectrogram where the time window of the analysis included the problematic period. To correct for this artefact conservatively, we interpolated the 40 Hz SNR time-course from -500 to 500 ms around physical PMD onset.

## Event-by-event image analysis of button press and SSVEP-SNR

Due to variations in the frequency and duration of PFI per participant, averaging data over participants is not straightforward. To resolve this, we performed image-based event-by-event analyses [(Fujiwara et al., 2017)](https://paperpile.com/c/ROJwbu/vFLn) to investigate whether the amount of PFI reported may reflect changes in log(SNR). Within each participant, all PFI events were sorted in descending order based on the sum of buttons pressed at each time point, and over a 2.5 second time window (detailed below) per disappearance/reappearance event. For this analysis, we counted three button presses as 3 even though participants might have tried to press 4 buttons. For PFI disappearances and reappearances, we averaged this over [0, +3] seconds and [-3, 0] seconds with respect to the button press or release, respectively. We call this sum of the number of buttons pressed over these time periods "the amount of PFI". We then resampled along the trial dimension to 100 samples to map from 0 to 1 (normalized event count) for each participant. Participant data was then smoothed along the normalized trial dimension and averaged across participants, to visualize the time-course of SNR as a function of normalized PFI. This resampling, smoothing and averaging process performed on button-press responses was repeated for the event-by-event time course of log(SNR), with the order of events predetermined by the corresponding button-press responses per participant. A schematic pipeline for this entire procedure is displayed in Supplementary Figure 2.

To quantify the relationship between log(SNR) and the amount of PFI, we grouped events when the amount of PFI was between 0 and 1, 1 and 2, or greater than 2. A median split based on the amount of PFI resulted in similar data and subsequent conclusions. The results of this event-by-event image analysis are displayed in Figure 4.


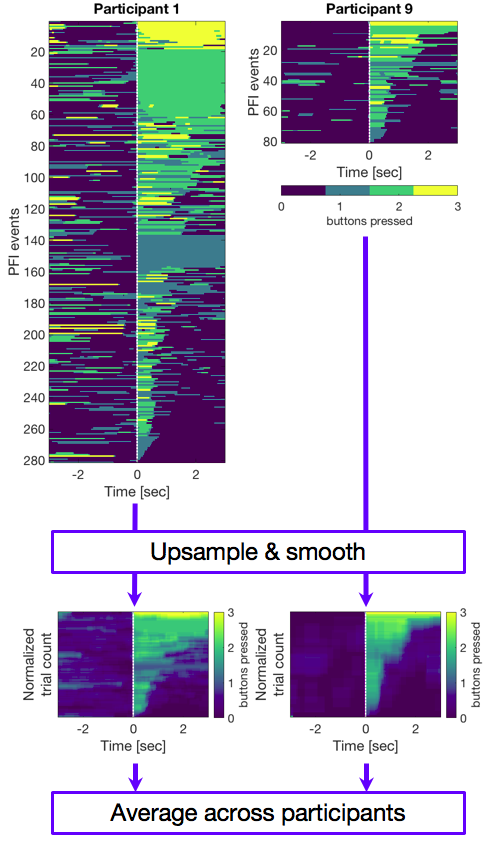


#### Supplementary Figure 2. Preprocessing for event-by-event based image analyses. PFI events were first sorted according to "the amount of PFI" (the sum of buttons pressed over 2.5 seconds) occurring after a button-press, or before a button-release event. Each image along the y-axis was then resampled to normalize the trial number into arbitrary units of 100 samples. A 15-sample moving average was then applied to smooth each image along the normalized event-dimension, before averaging across participants. The same process was also applied to RESS log(SNR) after sorting by the amount of PFI per event based on button-press (or -release) events. This image-based analysis enables us to compare PFI dynamics despite differences in the number of PFI events per participant.

## Reconstruction analysis to compare the impact of multiple-target disappearances and reappearances on SNR, during PFI and PMD

Due to our novel multiple-response task design which employs multiple targets, it is necessary to account for whether the temporal dynamics of log(SNR) during PFI or PMD differ due to the involvement of unique mechanisms, or are attributable to differences in the requirements to report on overlapping events. For example, in our data the increase of log(SNR) at PFI onset appears to be transient, and prior to subjective report, while changes during PMD occur later and are more sustained (Figure 5). Could these differences be due to unique neural substrates, or to the overlapping influence of temporally proximal PFI events? This is particularly important as the temporal profile of PFI and PMD may differ based on the way we programmed PMD; PFI events can accumulate for multiple targets in close temporal proximity, yet multiple PMD share a common temporal onset, even when spatially distributed. We approached this problem by performing an SNR reconstruction procedure, to model and compare the expected temporal dynamics of log(SNR) during PFI and PMD when accounting for accumulating disappearance and reappearance events. We hypothesised that if the same neural substrates were involved in PFI and PMD, then we should be able to predict one from the other, via our reconstruction procedure. This analysis progressed through three steps (Supplementary Figure 3).

####
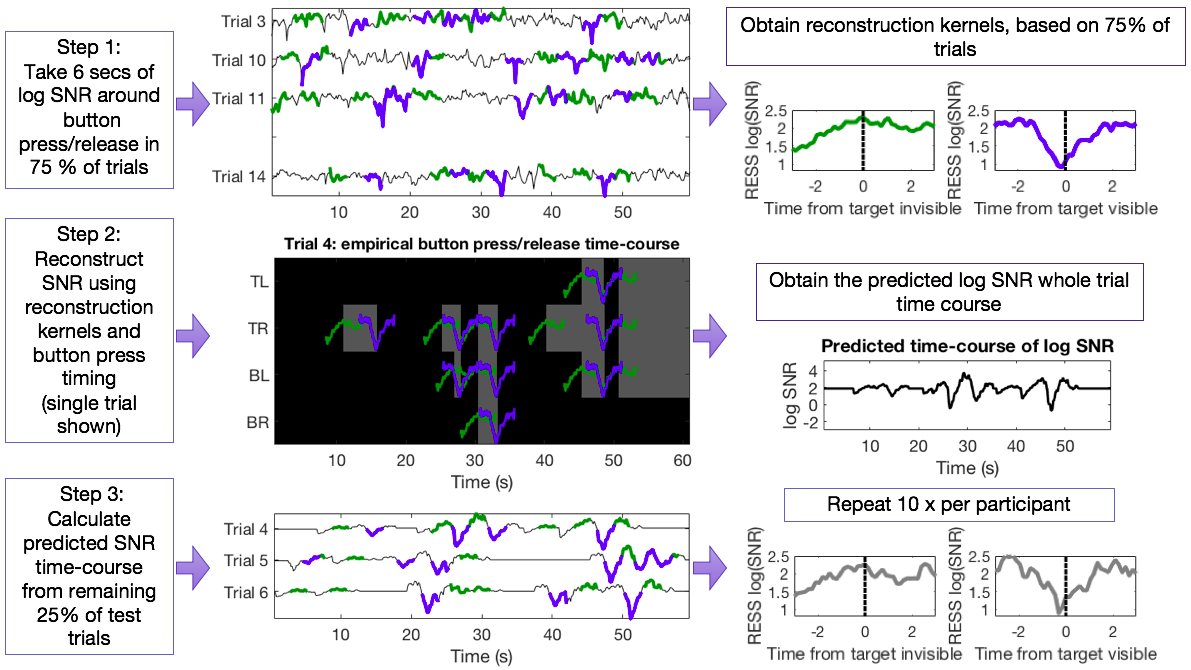


#### Supplementary Figure 3. Pipeline for SNR reconstruction analysis to estimate the impact of accumulated PFI disappearances/reappearances on the observed time course of log(SNR). Step 1: we first calculated the reconstruction kernels in response to target disappearance and reappearance events from 75% of training trials per participant. Log(SNR) around button press/release events (epoched -3 to +3 seconds) is shown in green/blue, respectively. Reconstruction kernels are computed as the mean log(SNR) time course around button press/release events (over 18 trials for this participant who had no rejected trials). Step 2: to predict the time course of log(SNR), we convolved the reconstruction kernels from Step 1 with recorded time of button press and release events in the remaining test trials (here only displaying 1 trial for demonstration purposes). As multiple PFI disappearances and reappearances can happen across target locations in close temporal proximity (< 1 second), this analysis enabled an estimation of the impact that consecutive PFI events have on SNR time course. The predicted time courses (gray) are computed as the mean log(SNR) during PFI events for test trials (over 6 trials for this participant). The predicted time courses are compared with the observed time courses from the same test trials (6 trials). This entire procedure was repeated 10 times per participant to obtain the mean predicted and observed time course for correlation analysis.

First, we calculated the mean log(SNR) time-course for PFI disappearances and reappearances using 75% of trials. Within these trials, we stepped through each time-point in the accumulative button-press responses (0-3 buttons pressed), and epoched the log(SNR) time-course from -3 to +3 seconds around the time of PFI events, which we defined as any change in button-press state (6-second epoch). For this analysis, we did not distinguish the number of disappearing targets at each time-point, just the direction of change (disappearing or reappearing), and obtained the mean disappearance/reappearance time courses which we subsequently used as reconstruction kernels. Second, using these reconstruction kernels, we then predicted the SNR in the remaining 25% of 60-second test trials. We did this by assuming linearity and time invariance in PFI responses, and predicted the 60-second whole-trial SNR time course by convolving the 6-second reconstruction kernels with the actual button-press or -release event times in the test trials. Outside of button press periods, we set the default SNR value as the baseline SNR value from the same trial (e.g. log(SNR) = 2.1 above). Third, from the reconstructed 60-second time course of SNR, we epoched from -3 to +3 seconds around the PFI events and obtained the mean predicted log(SNR) time course. Supplementary Figure 3b shows this procedure for one 60-second trial. We reconstructed a mean predicted SNR from across test trials, separately for PFI disappearance and reappearance. We repeated this reconstruction 10 times to obtain the mean predicted SNR per participant, which we then averaged across participants. We compared this measure to the observed mean log(SNR) time course from the same test trials.

We repeated the same procedure to compare the predicted SNR from PFI reconstruction kernels to the observed SNR during PMD. This was necessary due to the embedding of PMD within multi-target PFI, as PMD would often overlap with ongoing button-press and -release events signifying genuine PFI. We were then able to statistically determine whether the SNR time courses during PFI and PMD disappearances/reappearances were statistically distinct, by convolving the reconstruction kernels based on (training) PFI with the button-press or -release event times of (test) PFI and PMD.

To compare the predicted and the observed SNR time course, we evaluated the degree of correlation between them over the 6 seconds surrounding button-press and release, obtaining R^2^ for each individual participant. For the statistical analysis, we used repeated measures two-way ANOVA, testing the main effects of background harmonics (1f = 20Hz vs 2f = 40Hz) and the nature of disappearance/reappearance (PFI vs PMD) on the R^2^ between the observed and the predicted SNR time course. The results of this reconstruction analysis are displayed in Figure 5, and comparison of model fit is shown in Tables 1 and 2.

##

##

# Supplementary Analyses:


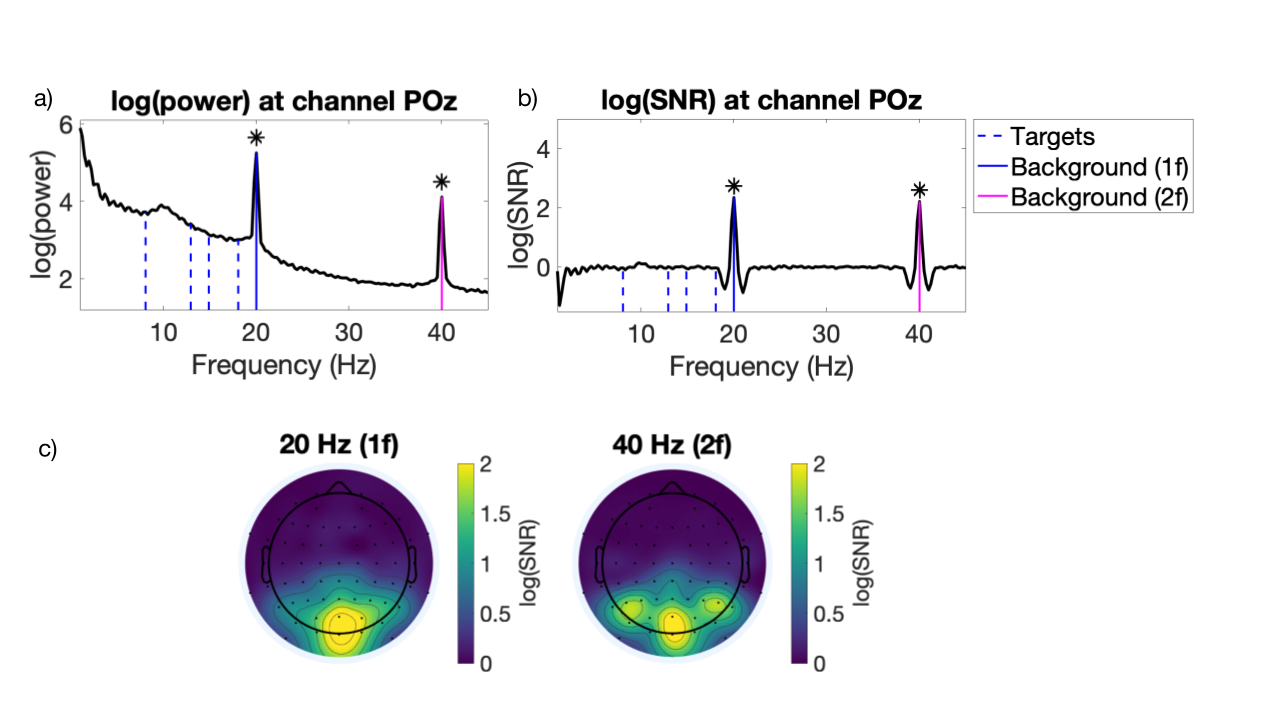


#### Supplementary Figure 4. Average SSVEP responses in our paradigm. a) The mean log(power) spectrum, and b) log(SNR) over all participants and periods of PFI at channel POz. Asterisks mark peaks significantly different from 0, FDR-adjusted across all frequencies to *p* < .05.

## Frequency-tagging during PMD

Having identified the successful entrainment of background responses (Supplementary Figure 4), we analyzed the time course of changes to the RESS log(SNR), focusing on 1f and 2f during PMD. As SSVEPs tend to be weak for peripherally presented stimuli [(Norcia et al., 2015)](https://paperpile.com/c/ROJwbu/dz6d), we checked if the physical removal of targets was strong enough to alter the time course of the RESS log(SNR). During PMD, we compared the mean RESS log(SNR) during -2 to -0.1 to +0.1 to +2 seconds (two-tailed paired samples *t*-tests). The SNR to background flicker increased upon target removal (1f, *t*(21) = 3.80, *p* = .0011; 2f, *t*(21)= 2.21, *p* = .038). The background SNR also decreased upon target return (1f, *t*(21)= -3.51, *p* = .0021; 2f, *t*(21) = -3.50, *p* = .0021). The increase/decrease of the RESS log(SNR) started upon button press/release (Supplementary Figure 5). These results are consistent with an interpretation that the background 1f and 2f SNR increases when peripheral regions are physically interpolated by the flickering background display.


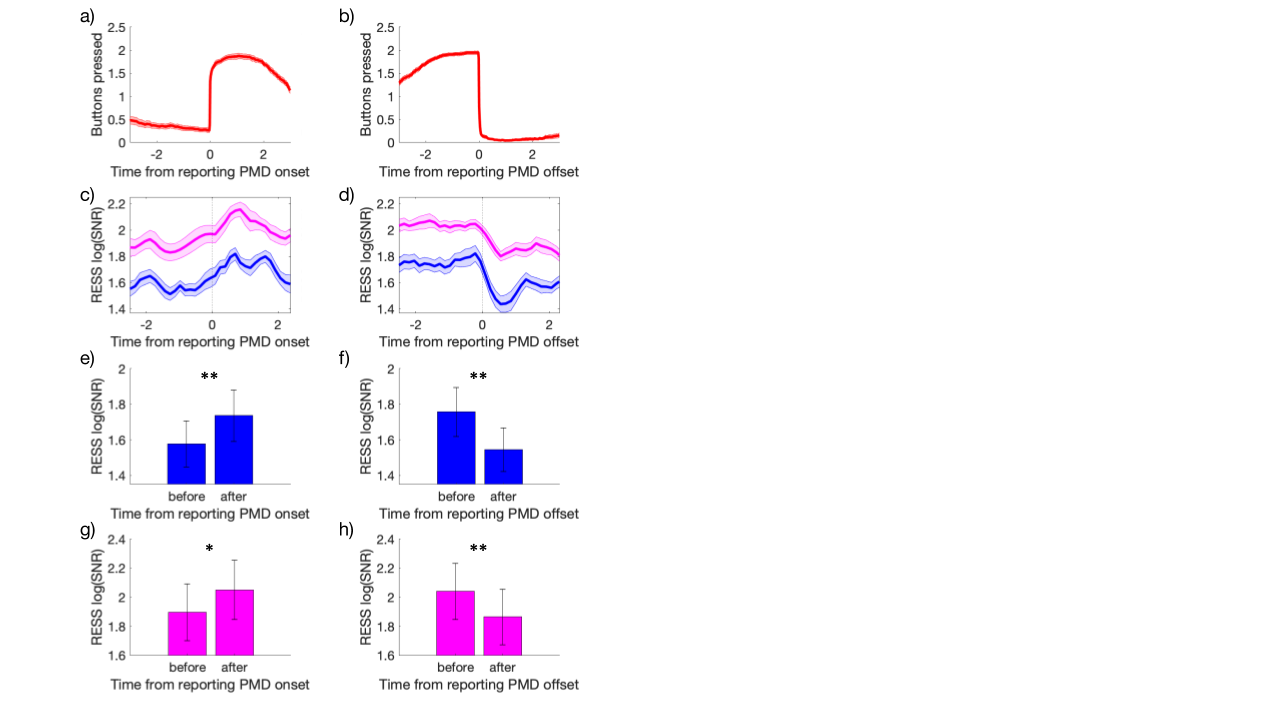


#### Supplementary Figure 5. Button press time course and background RESS log(SNR) around PMD. a-b) mean (± 1 SEM) button-press time course across participants when responding to the physical removal of targets near the onset (a) and the offset (b) of PMD. c-d) RESS log(SNR) for background SSVEP at 1f (20 Hz; blue) and 2f (40 Hz; magenta). Shading represents ± 1 SEM corrected for within-participant comparisons [(Cousineau, 2005)](https://paperpile.com/c/ROJwbu/l2Pk). e-h) Bar-charts for the statistical comparisons reported in text, comparing RESS log(SNR) before and after button press during PMD.

## Timing analysis: Jackknife procedure

To confirm the difference in latency between 1f and 2f responses prior to the subjective report of PFI, we also performed a non-parametric jackknife resampling procedure to estimate the standard error of the difference between crossover timepoints across participants [(Kohler, Cottereau, & Norcia, 2018)](https://paperpile.com/c/ROJwbu/EVxJ). Specifically, we calculated the difference in 1f and 2f crossover timepoints after leaving each participant out on a single permutation. We then estimated the standard error of all crossover differences in our *N*-1 subsets over all participants (using Equation (2); of [(Miller, Patterson, & Ulrich, 1998)](https://paperpile.com/c/ROJwbu/iDvM), and then derived a *t*-statistic from this estimate (Equation (3); [(Miller et al., 1998)](https://paperpile.com/c/ROJwbu/iDvM). This procedure confirmed that the difference between 1f (*M=* - 0.94, *SD* = 0.018 seconds) and 2f (*M*= -1.18, *SD* =0.03 seconds) crossover points was also statistically significant (*t*(21)= -1.81, *p* = .043).


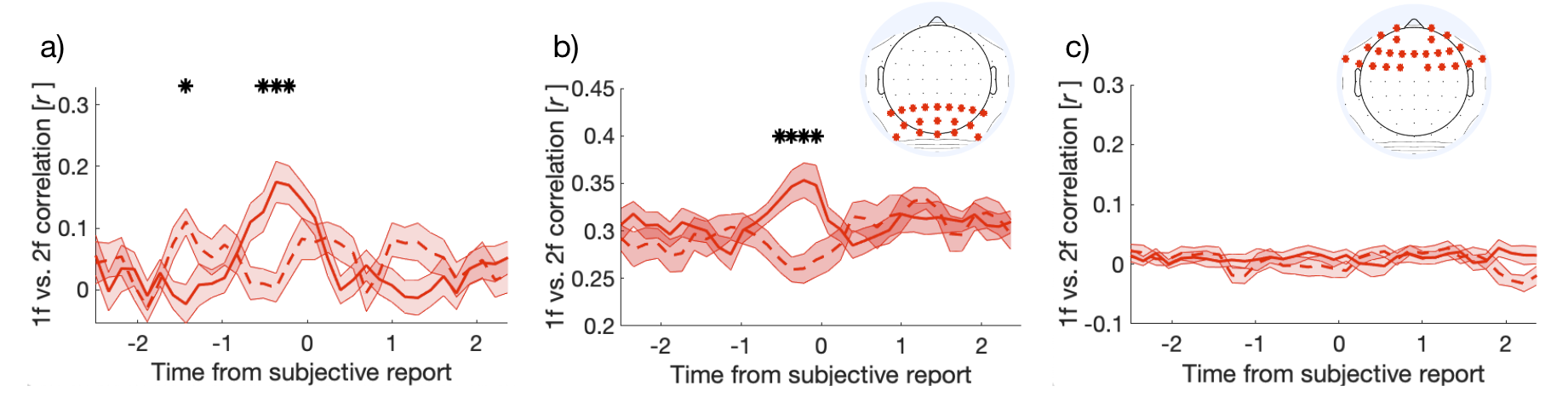


Supplementary Figure 6. Spatial correlation coefficient (r) between 1f and 2f (non-RESS) log(SNR) over time. Correlation between channels is shown for target disappearance (solid) and reappearance (broken lines). An increase in spatial-correlation, consistent with Figure 5, is shown when a) normalizing by the within channel SNR, and b) subselecting parieto-occipital electrodes. c) No change in correlation is shown within fronto-temporal electrodes. Asterisks denote time-points with significantly different correlation coefficients, comparing disappearance to reappearance (paired t-tests, cluster corrected). Shading reflects the SEM across participants corrected for within-subject comparisons.
